# Optimization of Protein Isolation and Label-Free Quantitative Proteomic Analysis in Four Different Tissues of Korean Ginseng

**DOI:** 10.3390/plants10071409

**Published:** 2021-07-09

**Authors:** Truong Van Nguyen, So-Wun Kim, Cheol-Woo Min, Ravi Gupta, Gi-Hyun Lee, Jeong-Woo Jang, Divya Rathi, Hye-Won Shin, Ju-Young Jung, Ick-Hyun Jo, Woo-Jong Hong, Ki-Hong Jung, Seungill Kim, Yu-Jin Kim, Sun-Tae Kim

**Affiliations:** 1 Department of Plant Bioscience, Life and Industry Convergence Research Institute, Pusan National University, Miryang 50463, Korea; ng.truong.win@gmail.com (T.V.N.); kimsso12@nate.com (S.-W.K.); min0685@gmail.com (C.-W.M.); 7012nn@naver.com (G.-H.L.); com5222@naver.com (J.-W.J.); rathi.divya71@gmail.com (D.R.); angel981123@naver.com (H.-W.S.); xb01xb@naver.com (J.-Y.J.); 2 Department of Botany, School of Chemical and Life Science, Jamia Hamdard, New Delhi 110062, India; ravigupta07@ymail.com; 3 Department of Herbal Crop Research, Rural Development Administration, Eumseong 27709, Korea; intron@korea.ac.kr; 4 Graduate School of Biotechnology and Crop Biotech Institute, Kyung Hee University, Yongin 17104, Korea; hwj0602@nate.com (W.-J.H.); khjung2010@khu.ac.kr (K.-H.J.); 5 Department of Environmental Horticulture, University of Seoul, Seoul 02504, Korea; ksi2204@uos.ac.kr; 6 Department of Life Science and Environmental Biochemistry, Life and Industry Convergence Research Institute, Pusan National University, Miryang 50463, Korea

**Keywords:** label-free proteomics, *Panax ginseng*, ginsenosides, cytochrome p450, UDP-glycosyltransferase, MEP pathway, MVA pathway, TCA/acetone, methanol/chloroform

## Abstract

Korean ginseng is one of the most valuable medicinal plants worldwide. However, our understanding of ginseng proteomics is largely limited due to difficulties in the extraction and resolution of ginseng proteins because of the presence of natural contaminants such as polysaccharides, phenols, and glycosides. Here, we compared four different protein extraction methods, namely, TCA/acetone, TCA/acetone–MeOH/chloroform, phenol–TCA/acetone, and phenol–MeOH/chloroform methods. The TCA/acetone–MeOH/chloroform method displayed the highest extraction efficiency, and thus it was used for the comparative proteome profiling of leaf, root, shoot, and fruit by a label-free quantitative proteomics approach. This approach led to the identification of 2604 significantly modulated proteins among four tissues. We could pinpoint differential pathways and proteins associated with ginsenoside biosynthesis, including the methylerythritol 4–phosphate (MEP) pathway, the mevalonate (MVA) pathway, UDP-glycosyltransferases (UGTs), and oxidoreductases (CYP450s). The current study reports an efficient and reproducible method for the isolation of proteins from a wide range of ginseng tissues and provides a detailed organ-based proteome map and a more comprehensive view of enzymatic alterations in ginsenoside biosynthesis.

## 1. Introduction

Ginseng (*Panax ginseng*) is a precious medicinal plant exhibiting significant economic values and pharmacological effects [1,2]. Owing to the presence of various bioactive compounds such as saponins, alkaloids, polysaccharides, free amino acids, and (poly)phenolics, ginseng has been proved to combat stress, improve the immune system, and maintain optimal oxidative status against aging, as well as assisting medical treatments related to central nervous system disorders, liver diseases, cardiovascular diseases, and cancer [1,3]. The world market of ginseng root and related products is worth USD 2084 million, suggesting a huge production and demand for ginseng products [2], and therefore, multiple studies at the genome [4], transcriptome [5], and metabolite [5] level have been conducted to understand the biology of this plant.

In addition, efforts have also been made to improve our understanding of ginseng at the protein level by utilizing proteomics approaches. Studies have focused on identifying stress-responsive and ginsenoside biosynthesis-related proteins, while some studies have concentrated on comparing and analyzing proteins from different ginseng parts and species [5,6,7,8]. However, a number of these studies used one or two tissues and were based on two-dimensional gel electrophoresis (2-DE) analysis, limiting the comprehensiveness of their proteome data [5,6,9]. Therefore, a systematic proteomics study using a wide range of tissues is necessary to provide a deeper understanding of ginseng.

Protein purification is a crucial step of the sample preparation, guaranteeing sufficient and high-quality proteins for proteome analysis [10]. TCA/acetone, phenol methanol, and methanol/chloroform precipitation methods have been developed for the isolation of plant proteins due to their efficiency in precipitating proteins and simultaneously removing interfering compounds [11]. A recent review [12] suggested that TCA/acetone precipitation displays high efficiency in the isolation of total proteins from a diversity of plant tissues while the phenol/methanol method effectively produces high-quality protein samples; minimizes protein degradation; and removes polysaccharides, ions, and nucleic acids. Besides, a study by Wessel and Flügge [13] pointed out that the methanol/chloroform precipitation can work well with different kinds of proteins, especially hydrophobic proteins, in the presence of detergents and with dilute samples. However, no single extraction method can reap the entire proteomes of a tissue or a plant species. Therefore, the combination of two or more approaches to integrate the strengths of each one for the isolation of proteins has been suggested [8,14]. A recent study by Wu [14] presented a protocol that was the combination of TCA/acetone precipitation and phenol extraction for the successful isolation of proteins from various recalcitrant tissues.

Advancements in proteomics approaches have facilitated the proteome analysis of various plants; however, difficulties in extracting relatively pure ginseng proteins have remained a primary obstacle [15]. Up to now, TCA/acetone method has been extensively used for extracting total ginseng proteins [7] while TCA precipitation and phenol extraction have been moderately employed to isolate ginseng proteins for 2-DE analysis [16]. Nonetheless, the efficiency of these methods has been tested on one or two ginseng tissues only, hindering their wide acceptability in ginseng proteome analysis [7,17,18]. Therefore, the development of a universal ginseng protein isolation method is a prerequisite for high-throughput ginseng proteome analysis.

**Figure 1:**
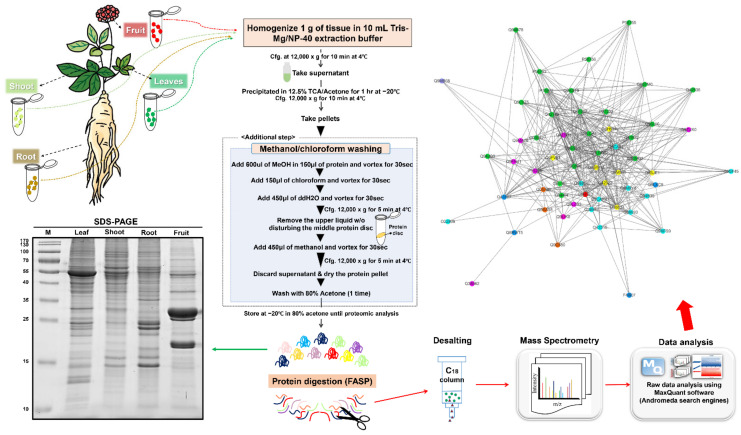
Workflow of the experiment. Ginseng samples were collected and homogenized in Tris–Mg/NP-40 buffer. After centrifugation at 16,000× *g* for 10 min at 4 °C, the supernatant was precipitated in 12.5% TCA/acetone at 4 °C for one hour. Protein pellets, obtained through centrifugation at 16,000× *g* for 10 min at 4 °C, were subsequently washed with methanol/chloroform, followed by trypsin digestion using the FASP method. The digested peptides were desalted and analyzed using a label-free quantitative proteomic approach. The obtained data were analyzed and annotated using MaxQuant, Perseus, and MapMan.

Here, an attempt was made to first evaluate the efficiency and reproducibility of different protein extraction methods, namely TCA/acetone, TCA/acetone–MeOH/chloroform, phenol–TCA/acetone, and phenol–MeOH/chloroform, followed by utilizing the most effective approach for the comparative proteome analysis (Figure S1). Moreover, an attempt was also made to generate a relatively comprehensive proteome map of ginseng fruit, leaf, root, and shoot using a label-free quantitative proteomics approach (Figure 1). Furthermore, through the significantly modulated proteins, we generated a more comprehensive view of the ginsenoside biosynthesis. This in-depth study provides new insights into the protein complement of different ginseng tissues.

## 2. Results and Discussion

### 2.1. Optimization of Ginseng Protein Extraction Method

The medical value of *Panax ginseng* increases with its age, but for its use as medicine and commercial production, a growth period of 4–6 years is often required [18]. Therefore, in order to meet the practicality and enhance the reliability of the current study, fruit, leaf, root, and shoot samples were harvested from various 4-year-old *Panax ginseng* plants and pooled together before analysis. As ginseng leaves contain various natural contaminants such as lipids, saccharides, and various photosynthetic pigments, the extraction of proteins from ginseng leaves is more challenging than from other ginseng parts [19]. Therefore, we used ginseng leaves as a model sample for checking the protein extraction efficiency of four different extraction methods, namely TCA/acetone, TCA/acetone–MeOH/chloroform, phenol–TCA/acetone, and phenol–MeOH/chloroform (Figure S1). Eliminating interfering compounds is an initially crucial step in extracting proteins from plant samples. A review by Wu [12] revealed that finely powdered plant samples can be directly subjected to TCA/acetone but not to phenol. Therefore, to ensure the homogeneity of the samples, the finely ground ginseng samples were first homogenized with Tris–Mg/NP-40 extraction buffer, and the OS was subsequently extracted using four different abovementioned methods.

**Figure 2:**
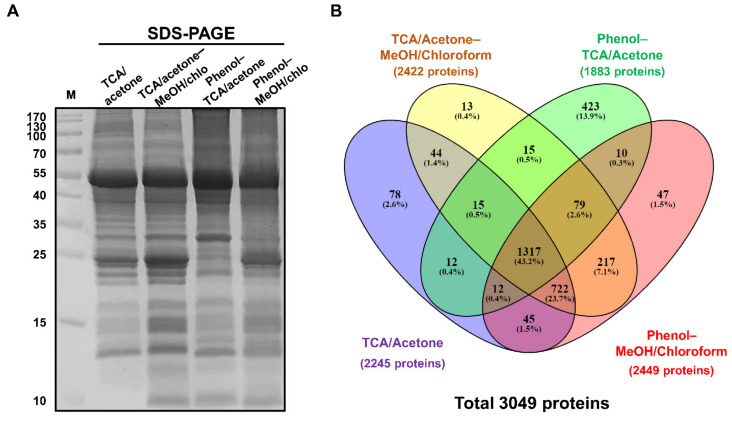
SDS-PAGE of proteins isolated from ginseng leaf using TCA/acetone, TCA/acetone–MeOH/chloroform, phenol–TCA/acetone, and phenol–MeOH/chloroform methods (**A**). Venn diagram showing the distribution of proteins isolated from ginseng leaves using four different protein extraction methods (**B**).

SDS-PAGE analysis of isolated proteins showed that using the TCA/acetone–MeOH/chloroform method produced more protein bands with a high resolution on the gel than the other tested methods (Figure 2A). Furthermore, the label-free quantitative proteomic analysis led to the identification of 36,145 peptides, corresponding to 4705 protein groups. The average numbers of peptides and unique peptides were 20,383 and 8256, 22,552 and 8919, 22,437 and 7919, and 22,437 and 8981 for TCA/acetone, TCA/acetone–MeOH/chloroform, phenol–TCA/acetone, and phenol–MeOH/chloroform, respectively (Table S1; Figure S2A). The average sequence coverage was 13.24, 14.99, 16.40, and 15.26 (%) for TCA/acetone, TCA/acetone–MeOH/chloroform, phenol–TCA/acetone, and phenol–MeOH/chloroform, respectively (Table S1; Figure 2B). Filtering out by applying a cut-off value of 75% within three technical replicates of each sample led to the identification of 3049 proteins (Figure 2B), of which 2449, 2422, 2245, and 1883 proteins were identified when using phenol–MeOH/chloroform, TCA/acetone–MeOH/chloroform, TCA/acetone, and phenol–TCA/acetone extractions, respectively (Table S1; Figure 2B). Isoelectric point (Figure S2C), molecular weight (Figure S2D), and hydrophobicity (GRAVY) (Figure S2E) of most of these proteins were between 20 and 160 kDa, 4 and 12, and −2 and 1, respectively. Subcellular prediction analysis using CELLO2GO web-based software showed a relatively similar distribution of proteins isolated using the four different methods over 11 locations (Figure S2F). Since the numbers of proteins identified by each method were relatively similar, there was not a large difference in the molecular weight, isoelectric point, and hydrophobicity of proteins among the tested approaches. 

Common methods based on TCA/acetone precipitation and phenol extraction, which have successfully isolated ginseng proteins from one or two ginseng tissues for 2-DE analysis [17], might be no longer effective in extracting a wide range of ginseng tissues for label-free quantitative proteomic analysis. Alternatively, the idea of combining two extraction methods to incorporate the strengths of every single one for isolating proteins from different ginseng tissues has shown considerable potential. Particularly, a recent study by Li [8] showed that the combination of GdnHCl with methanol/chloroform precipitation led to improved extraction of proteins from ginseng cauline leaves, compared with GdnHCl lysate and Tris–HCl lysate methods. However, this combination still displayed certain limitations as the SDS-PAGE quality and the number of identified proteins were relatively modest [8]. In the current study, the TCA/acetone–MeOH/chloroform method maintained the advantages of both TCA/acetone precipitation, which allows extraction of total proteins [20], and MeOH/chloroform extraction, which efficiently removes remaining contaminants (especially lipids) without clear quantitative loss of proteins [13], resulting in a better extraction of ginseng proteins as observed on the SDS-PAGE (Figure 2A) and by the number of identified proteins (Figure 2B). An extraction method is considered to be effective when it reproducibly attains the most comprehensive proteome and simultaneously minimizes protein degradation and contaminants [20]. Therefore, although the phenol–MeOH/chloroform method produced a slightly higher number of identified proteins than the TCA/acetone–MeOH/chloroform, the poor gel profile and high toxicity to humans of phenol made it an unsuitable choice for our subsequent analysis. The TCA/acetone–MeOH/chloroform could produce a clear gel profile and a higher number of identified proteins, compared with the other tested methods; therefore, it was utilized to extract proteins from ginseng tissues for global identification.

### 2.2. Label-Free Quantification Using Four Different Ginseng Tissues

**Figure 3:**
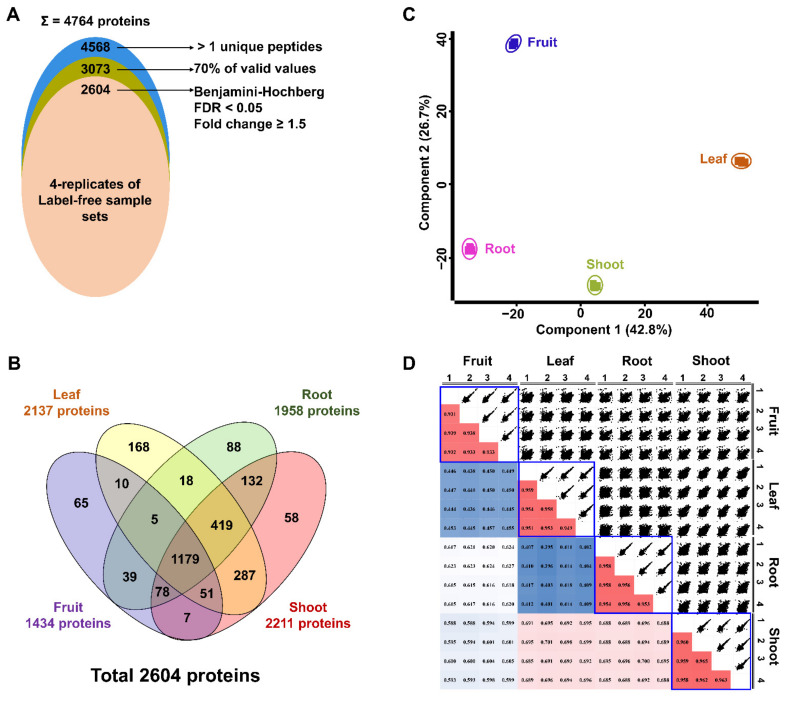
A total of 4764 protein groups were identified in this study. Out of these, 2604 proteins were significantly modulated among four tissues (**A**). Venn diagram showing the distribution of 2604 proteins (**B**). Principle component analysis of the differentially regulated proteins (**C**). Multi-scatter plots of label-free protein intensities between different technical replicates of the samples with Pearson correlation coefficient values (**D**).

The LC-MS/MS analysis led to the identification of a total of 39,275 peptides, which corresponded to 4764 protein groups. A cut-off value of 75% was applied within four technical replicates of each tissue sample, leading to the identification of 3073 proteins (Figure 3A). Of these, 1434, 1958, 2137, and 2211 proteins were found to be in the fruit, root, leaf, and shoot samples, respectively. Subsequently, multiple ANOVA tests, controlled by Benjamini–Hochberg FDR threshold of 0.05, were applied on the identified proteins to demarcate 2604 differentially regulated proteins with fold change more than 1.5 (Table S2; Figure 3B). While 1179 proteins were common in all four tissues, 287, 18, 132, and 39 proteins were common in the leaf/shoot, leaf/root, shoot/root, and root/fruit samples, respectively (Figure 3B).

Sequential multi-scatter plot and principal component analysis (PCA) were thereafter performed to analyze the correlation and variations among the four ginseng tissues (Figure 3C,D). The PCA plot illustrates a clear separation among all of the four sample sets, demarcating the distinctness of the differential tissue proteomes (Figure 3C). While the root and leaf samples were separated in PC1 accounting for 42.8% of the total variation, the shoot and fruit samples were resolved in PC2 that accounted for 26.7% of the total variation. Furthermore, the multi-scatter plot with the Pearson correlation coefficients of the technical replicates in each sample set ranging from 0.931 to 0.965 indicated a strong correlation among the technical replicates of the same samples (Figure 3D).

Previously, ginseng proteomic studies were based primarily on 2-DE analysis, leading to the identification of a relatively low number of proteins (about 1000 proteins) in these studies [6,16]. The development of the shotgun techniques, coupled with advancements in MS, has significantly improved the number of proteins identified from various plant tissues [15]. A recent study combined GdnHCl with methanol/chloroform precipitation to extract proteins from ginseng cauline leaves, leading to the identification of 1366 proteins [8]. However, by applying basic fractionation, the number of proteins isolated using this method increased significantly to 3608 proteins [8]. In the current study, by using the TCA/acetone–MeOH/chloroform for protein extraction, followed by a label-free quantitative proteomic analysis, we successfully identified 4764 proteins from ginseng fruit, leaf, root, and shoot (Figure 3A). This is the first study on ginseng in which such a high number of identified proteins is reported from a wide range of tissues using only one extraction method without fractionation. However, further investigations comparing this method with different MS sample preparations such as single-pot solid-phase-enhanced sample preparation (SP3) [21], in-StageTip digestion (iST) [22], and the suspension trapping (S-Trap) filter [23] using various ginseng tissues might provide a deeper understanding of the sample preparation for ginseng proteomics.

### 2.3. Functional Classification of Identified Proteins

#### 2.3.1. Functions of Commonly Identified Proteins among Four Tissues

**Figure 4:**
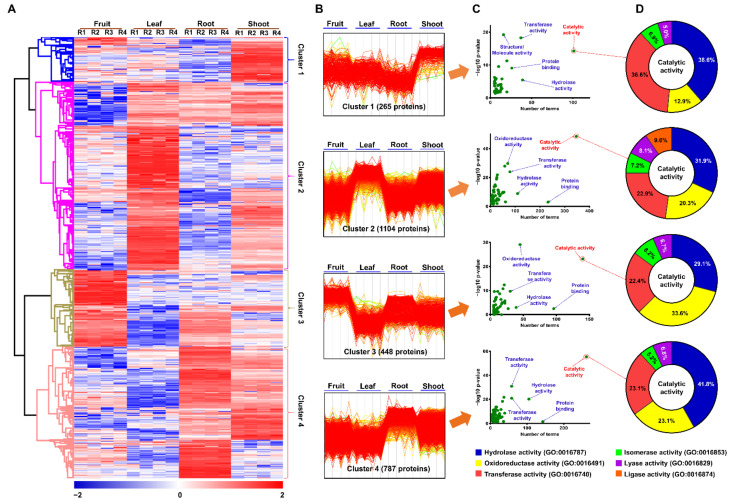
Expression profile of 2604 significantly modulated proteins identified by label-free quantitative proteome analysis. Hierarchical clustering (**A**) was carried out by Perseus software. Expression patterns of 4 protein clusters (**B**). Gene ontology analysis was performed for functional annotation of proteins in four clusters using AgriGO (ver. 2.0) (**C**,**D**).

For the further investigation of the significantly modulated proteins, we performed hierarchical clustering analysis (HCA) which separated all the identified proteins into four clusters based on log_2_ of the z-score normalized intensities among the technical replicates of each sample (Figure 4A). While Cluster 1 consisted of 265 proteins with high abundance in the shoot, Cluster 2 included 1104 proteins with increased abundance in the leaf. Clusters 3 and 4 contained 448 and 787 proteins, which were maximally accumulated in the fruit and root, respectively (Figure 4B)

For functional annotation of the identified proteins, we carried out gene ontology (GO) enrichment analysis via AgriGO through homolog identification of *P. ginseng* proteins in *A. thaliana* (TAIR10) [24] (Table S3). Notably, in the GO classification of molecular function, catalytic activity was the largest GO term in Clusters 1, 2, 3, and 4 with the involvement of 101 (38.1%), 347 (31.4%), 140 (31.3%), and 262 (33.3%) proteins, respectively (Figure 4C). Hydrolase activity, oxidoreductase activity, and transferase activity were the three main subgroups of catalytic activity found in all of the four clusters, while ligase activity was found in only Cluster 2 (Figure 4D). The metabolism overview of MapMan analysis indicated that most of the proteins related to the catalytic activity in Cluster 1 were involved in the biosynthesis of methionines, cellulose and precursors, phospholipids, flavonoids, and isoprenoids, which are more active in the shoot [25]. Meanwhile, the proteins associated with catalytic activity in Cluster 2 were majorly involved in the biosynthesis of various amino acids, photosynthesis, nucleotide metabolism (synthesis of purines and pyrimidines), CHO metabolism (synthesis of starch and sucrose), and the synthesis of secondary metabolites (flavonoids, isoprenoids, and phenylpropanoids), which take place predominantly in the leaf of plants [25]. By contrast, most of the proteins that belonged to the catalytic activity in Clusters 3 and 4 were mainly associated with the degradation of different molecules (such as amino acids, nucleotides, lipids, starch, sucrose, and flavonoids), glycolysis, and tricarboxylic acid cycle, which commonly occur in the fruit and root of plants [25] (Table S3). The result of MapMan analysis is consistent with the result from the HCA (Figure 4A,B).

#### 2.3.2. Functions of Tissue-Specific Proteins

**Figure 5:**
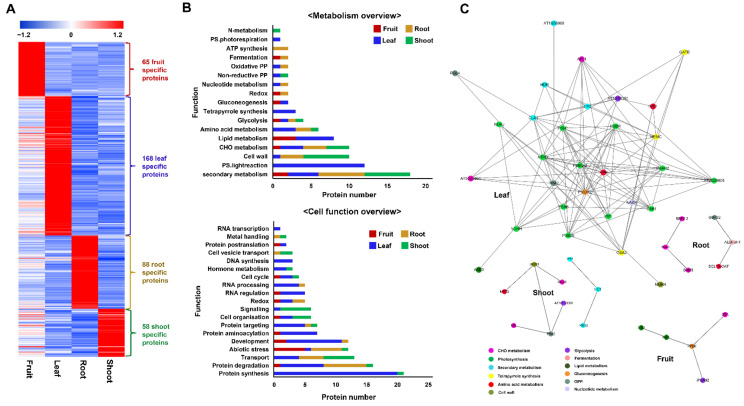
Overview of tissue-specific proteins (**A**). Functional annotation of tissue-specific proteins was carried out using MapMan (**B**). Protein–protein interaction networks of tissue-specific proteins related to metabolic processes were analyzed using STRING (ver. 11.0), coupled with Cytoscape (ver. 3.7.2) (**C**).

Among the 2604 identified proteins, 65, 168, 88, and 58 proteins were specifically identified in the fruit, leaf, root, and shoot, respectively (Table S2; Figure 5A). For understanding the functional significance of these proteins, the metabolic overview and cell function were analyzed using MapMan (Figure 5B), followed by an interactome analysis using STRING (v. 11.0) (Figure 5C).

The metabolism overview of MapMan analysis revealed that among 65 proteins specific to the fruit, 10 proteins were classified into six metabolic groups, of these, lipid metabolism was the largest group, containing acyl-(acyl-carrier-protein) desaturase and 3-ketoacyl-acyl carrier protein synthase I involved in the fatty acid synthesis and elongation (Table S4). For the leaf-specific proteins, 13 groups accounting for 37 proteins were categorized; of these, the photosynthesis process with proteins associated with the light reaction of photosystems I and II and photorespiration was the major metabolism. Regarding the 88 root-specific proteins, 10 metabolic groups accounting for 21 proteins were sorted, of which secondary metabolism was the largest, containing six proteins. Differently, cell wall with six proteins associated with the formation and modification of the cell wall was the most dominant metabolic group of shoot-specific proteins (Table S4).

Furthermore, the cell function of MapMan analysis showed that six groups accounting for 14 fruit-specific proteins were categorized, of these, abiotic stress was the largest group, with five proteins. Protein synthesis, protein aminoacylation, and protein targeting were the most dominant groups associated with 38 proteins specific to the leaf. Meanwhile, the largest groups of proteins specific to the root were protein degradation and biotic stress. Transport and signaling were the most predominant groups related to 10 proteins included exclusively in the shoot (Figure 5B).

To have a global view of all possible interactions among specific proteins that were involved in the metabolisms of each sample set, protein–protein interaction networks were created. After STRING functional enrichment analysis, a total of 5, 29, 6, and 9 proteins uniquely stemming from the fruit, leaf, shoot, and root, respectively, showed interactions on the network (Figure 5C). Among these, photosynthesis was the primary metabolism influencing various activities in the leaf, while CHO metabolism and secondary metabolism were predominant metabolisms in the root and shoot, respectively. Gluconeogenesis was the metabolism linked to different metabolic activities in the fruit.

Tissue-specific proteins are important factors contributing to differences in anatomical characteristics and physiological functions among living tissues. Therefore, some studies have been conducted to identify and characterize tissue-specific proteins in various plants [26,27]. On *P. ginseng*, few studies have determined proteins specific to ginseng leaves and roots. A study by Seung [28] successfully identified and characterized root-specific RNase-like proteins (GMPs) in roots of wild ginseng, which might work as vegetative storage proteins promoting its survival in the natural habitat. Furthermore, Li [8] highlighted 878 and 1754 proteins specific to the roots and cauline leaves, respectively. The author also revealed that the cauline leaf-specific proteins were primarily associated with photosynthesis and related energy conversion while the proteins specific to the root were involved in the biosynthesis and modification of biomacromolecules [8]. The functional annotation and molecular processes highlighted in the leaf and root in the current study are were relatively consistent with the previous report [8]. However, as the present study was performed on all fruit, leaf, root, and shoot tissues, the number of overlapped proteins was significantly increased, while the number of tissue-specific proteins was also highlighted.

### 2.4. Decoding the Proteome Modulations in Association with Ginsenoside Biosynthesis

Ginsenosides, a well-known triterpenoid saponin type in the ginseng plant, are natural secondary metabolites of ginseng, exhibiting a diversity of medicinal effects [1]. Recently, more than 180 ginsenosides have been identified and categorized into three main types: protopanaxadiol (PPD) type, protopanaxatriol (PPT) type, and oleanane type, with the first two commonly existing in *P. ginseng* [29,30]. The biosynthesis of ginsenosides can be divided into three main stages: (1) the biosynthesis of the precursor isopentenyl pyrophosphate (IPP) and dimethylallyl pyrophosphate (DMAPP) through the MVA and MEP pathways, (2) the conversion of IPP and DMAPP into 2,3-oxidosqualene, and (3) the formation of ginsenosides and sterols from 2,3-oxidosqualene [31].

**Figure 6:**
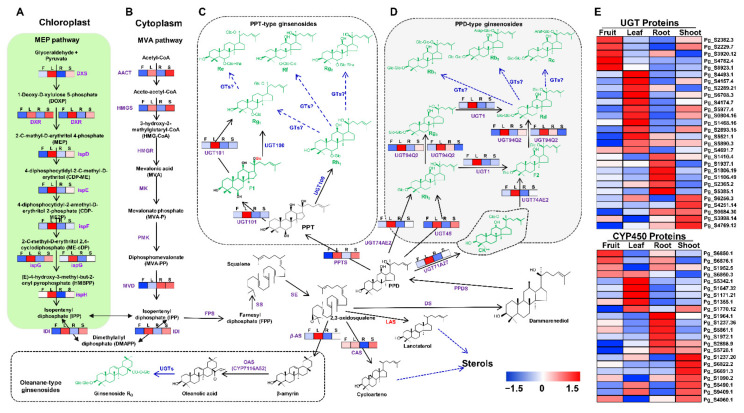
Expression profiles of identified proteins involved in the MEP (**A**) and the MVA (**B**) pathways. PPT-type (**C**) and PPD-type (**D**) ginsenosides. The abundance of UGTs and CYP450s related to ginsenoside biosynthesis (**E**). Color codes represent abundance patterns of identified proteins wherein red and blue indicate a high and low abundance of proteins in particular tissues, respectively. F—fruit, L—leaf, R—root, S—shoot.

It is a fact that ginsenosides are unevenly distributed in different parts of ginseng. A few studies have confirmed that the total ginsenoside content of the ginseng leaf and fruit was higher than that of the root [32], yet there have been no studies elucidating the molecular mechanism for this difference. This study, for the first time, revealed a relatively comprehensive proteome profile of the ginseng fruit, leaf, root, and shoot, providing a new understanding of the molecular basis for the variation in the ginsenoside content among the four tissues. Our result identified a total of 67 proteins associated with the ginsenoside biosynthesis (Table S5). Of these, acetyl-CoA C-acetyltransferase (ACCT), hydroxymethylglutaryl-CoA synthase (HMGS), and diphosphomevalonate decarboxylase (MVD) related to the MVA pathway were more abundant in the shoot. Nine proteins associated with the MEP pathway, namely one protein of 1-deoxy-D-xylulose-5-phosphate synthase family (DXS), two proteins of 1-deoxy-D-xylulose 5-phosphate reductoisomerase family (DXR), one protein of 2-C-methyl-D-erythritol 4-phosphate cytidylyltransferase family (ispD), one protein of 4-diphosphocytidyl-2-C-methyl-D-erythritol kinase family (ispE), one protein of 2-C-methyl-D-erythritol 2,4-cyclodiphosphate synthase family (ispF), two proteins of (E)-4-hydroxy-3-methylbut-2-enyl-diphosphate synthase family (ispG), and one protein of 4-hydroxy-3-methylbut-2-enyl diphosphate reductase family (ispH), showed higher abundance in the leaf. In addition, 28 UGTs were identified, of which 5, 12, 6, and 5 proteins were highly accumulated in the fruit, leaf, root, and shoot, respectively. Furthermore, 22 CYP450s were also identified, of which 4, 5, 6, and 7 proteins were highly abundant in the fruit, leaf, root, and shoot, respectively. Proteins such as isopentenyl-diphosphate delta-isomerase (IDI) and beta-amyrin synthase (β–AS) were also identified with high abundance in the ginseng leaf sample, while cycloartenol synthase (CAS) was highly accumulated in shoot and leaf samples. Besides, 2, 3, 6, and 1 proteins related to the biosynthesis of ginsenosides were found to be specific to the fruit, leaf, root, and shoot, respectively (Figure 6; Table S5).

Biosynthesis of IPP and DMAPP is essential to most living organisms. Depending on species, these precursors of isoprenoids can be synthesized through only the MVA pathway like some archaea and eukaryotes or only the MEP pathway like most bacteria or both of these pathways in most photosynthetic eukaryotes [33]. The MVA pathway is responsible for the conversion of acetyl-CoA into IPP and DMAPP, while the MEP pathway produces the IPP and DMAPP from glyceraldehyde and pyruvate (Figure 6A,B). In *P. ginseng*, studies based on phytochemical and inhibitor experiments, transcriptome, and genome sequencing revealed that the biosynthesis of IPP and DMAPP, the precursors of ginsenosides, has the involvement of both MVA and MEP pathways [24,31,34]. In addition, by conducting deep RNA sequencing on the 1–5-year-old ginseng root samples and five different tissues, Xue [35] not only determined most genes related to the MVA and MEP pathways but also pointed out the relative expression of these genes among different aging samples and tissues. However, these genes are not directly involved in the reactions of the MVA and MEP pathways, but their products (enzymes) are. This means that the abundance pattern of these enzymes in the fruit, leaf, root, and shoot of ginseng might be a deciding factor for the differences in the biosynthesis of the IPP and DMAPP and subsequently of tissue-specific CYP450s and UGTs, differentiating the types and concentration of ginsenosides in various parts of the ginseng plant [29,36]. In the present study, the higher abundance of proteins related to the MVA pathway (ACCT, HMGS, and MDV) was observed in the shoot, while all proteins associated with the MEP pathway (1 DXS, 2 DXR, ispD, ispE, ispF, 2 ispG, and ispH) showed the highest abundance in the leaf. These findings suggest that the biosynthesis of ginsenosides in the shoots might have the major involvement of the MVA pathway, while the biosynthesis of ginsenosides in the leaves might rely majorly on the MEP pathway. The findings are logically suitable to the plastic location of the MEP pathway and in concordance with the results from the research of Xue [35] that all genes related to the MEP pathway had higher levels of gene expression in the leaf than the root of *P. ginseng*.

The present study also highlights the increased abundance of cycloartenol cyclase (CAS) in the shoot and *β*-amyrin synthase (*β*-AS) in the leaf. The precursors, IPP and DMAPP, are converted into several metabolic intermediates and then to 2,3-oxidosqualene, which in turn undergoes variable cyclization by oxidosqualene cyclases (OSCs), hydroxylation by CYP450s, and glycosylation by UGTs to form an array of ginsenosides (Figure 6) [33]. The formation of sterols from 2,3-oxidosqualene is catalyzed by two different OSCs, namely lanosterol synthase (LAS) and CAS, while *β*-AS is involved in the production of oleanane. The formation of sterols from 2,3-oxidosqualene is catalyzed by two different OSCs, namely lanosterol synthase (LAS) and CAS, while *β*-AS is involved in the production of oleanane [29,37]. Our findings led to speculation that the biosynthesis of sterols might be more active in the shoot samples, whereas the pathways involving the production of the triterpenoid oleanane are differentially active in leaf tissues.

The PPD- and PPT-type saponins make up a majority of ginsenosides in *P. ginseng*. The biosynthesis of PPD- and PPT-type saponins occurs when 2,3-oxidosqualene is converted into dammolarenediol by dammolarenediol synthase (DS), then into protopanaxadiol (PPD) by cytochrome P450 CYP716A47 (PPDS) before undergoing one more hydroxylation catalyzed by cytochrome P450 CYP716A53v2 (PPTS) to form protopanaxatriol (PPT). The PPD and PPT are subsequently glycosylated by different UGTs to form a diversity of PPD- and PPT-type ginsenosides [33,35]. In our study, six UGTs related to the biosynthesis of PPD-type saponins were found to be differentially accumulated in the leaf tissues, including UGT71A27 (Pg_S6256.3) catalyzing the biosynthesis of compound K from PPD, UGT45 (Pg_S5977.4) converting PPD into Rh2, UGT47AE2 (Pg_S4174.7) catalyzing the biosynthesis of Rh2 from PPD and the biosynthesis of F2 from compound K, UGT94Q2 (Pg_S6708.3 and Pg_S2289.21) catalyzing the conversion of ginsenoside Rh2 to ginsenoside Rg3 and triggering the biosynthesis of ginsenoside Rd from ginsenoside F2, and UGT1 (Pg_S4493.1) triggering C20–OH glycosylation of ginsenoside Rg3 to produce ginsenoside Rd and converting Rh2 to F2 [38,39]. Besides, two proteins participating in the formation of PPT-type saponins comprising PPTS (Pg_S1770.12) catalyzing the formation of PPT from PPD and UGT101 (Pg_S4157.4) catalyzing the biosynthesis of ginsenoside F1 from PPT and the conversion of ginsenoside F1 to ginsenoside Rg1 also displayed a high abundance in the leaf samples [40]. The increased abundance of these proteins in the leaf demonstrated that the biosynthesis of PPD- and PPT-type ginsenosides in the leaf tissues is promisingly higher than in the fruit, root, and shoot. Furthermore, the appearance of CYP450s and UGTs specific to the fruit, leaf, root, and shoot may explain the existence of ginsenosides that are specific to each tissue. These results are consistent with a previous study by Kang [32] showing that the total ginsenoside content in the leaf of *P. ginseng* is 12 times higher than that of the main root and that the leaves of *P. ginseng* contain a high amount of ginsenosides Rh1 and Rb3, whereas its main roots have a higher quantity of ginsenosides Rb1 and Rc.

## 3. Materials and Methods

### 3.1. Plant Materials

*P. ginseng* cv. Chunpoong was grown in a controlled growth chamber at the Department of Ginseng Research, National Institute of Horticultural and Herbal Science (NIHHS), Rural Development Administration (RDA), Eumseong, Korea (latitude 36°94, longitude 127°75). The average temperature and humidity of the greenhouse were maintained at 22.5 ± 2.5 °C and 50 ± 10%, respectively. Four-year-old leaves, shoots, roots, and fruits from five different ginseng plants were harvested and immediately stored at −80 °C for analysis.

### 3.2. Total Protein Extraction

The leaf samples (1 g) of 4-year-old plants from five different plants were pooled together, homogenized in 10 mL of Tris–Mg/NP-40 extraction buffer (0.5 M Tris–HCl, 2% (*v*/*v*) NP-40, 20 mM MgCl_2_, 2% (*v*/*v*) *β*-mercaptoethanol, and 2% (*w*/*v*) polyvinylpolypyrrolidone, pH 8.3) and subjected to centrifugation at 16,000× *g* for 10 min at 4 °C. The OS was used for the subsequent extractions using trichloroacetic acid (TCA)/acetone, TCA/acetone–MeOH/chloroform, phenol–TCA/acetone, and phenol–MeOH/chloroform methods.

The TCA/acetone method was carried out as described previously [7,41]. Briefly, the OS was incubated with 4 volumes of 12.5% (*w*/*v*) TCA/acetone containing 0.07% (*v*/*v*) *β*-mercaptoethanol for 1 h at −20 °C and then centrifuged at 16,000× *g* for 10 min at 4 °C to obtain protein pellets. The TCA/acetone–MeOH/chloroform method was performed as described previously [13]. Briefly, the OS was first extracted using the TCA/acetone method, and the obtained proteins were then mixed with 4 volumes of methanol, then an equal volume of chloroform, and then 3 volumes of the sterile distilled water before being centrifuged at 16,000× *g* for 5 min to collect protein pellets. For the phenol–TCA/acetone, the OS was vigorously mixed with the same volume of saturated phenol and separated into two phases through centrifugation at 3500× *g* for 5 min at 4 °C. The lower phase containing proteins was incubated with 4 volumes of 12.5% (*w*/*v*) TCA/acetone containing 0.07% (*v*/*v*) *β*-mercaptoethanol for 1 h at −20 °C before being centrifuged at 16,000× *g* for 10 min at 4 °C to collect protein pellets. The phenol–MeOH/chloroform method was performed similarly to the phenol–TCA/acetone method with a slight difference: the lower phase yielded from the phenol extraction was incubated with 4 volumes of methanol and 1 volume of chloroform for 1 h at −20 °C prior to being centrifuged at 16,000× *g* for 10 min at 4 °C to collect protein pellets. The resulting pellets of these methods were finally washed with 80% acetone containing 0.07% (*v*/*v*) *β*-mercaptoethanol and then stored at −20 °C until further analysis.

The extraction of total proteins from ginseng fruits, leaves, roots, and shoots was conducted using the TCA/acetone–MeOH/chloroform as described above. 

### 3.3. Label-Free Quantitative Proteome Analysis Using Q-Exactive Mass Spectrometer

Label-free quantitative proteomic analysis of ginseng fruit, leaf, root, and shoot samples was performed as described previously [7]. Briefly, the digested peptides, obtained from the in-solution trypsin digestion using the FASP method, coupled with a 30k spin filter (Merck Millipore, Darmstadt, Germany) [42], were desalted using C_18_ column (Oasis HLB 1 cc Vac Cartridge, 30 mg sorbent per cartridge, 30 µm, 100/pk, WAT094225, Waters, Ireland). Subsequently, the desalted peptides were dissolved in solvent A (water/ACN, 98:2 *v*/*v*; 0.1% formic acid), followed by the reversed-phase chromatography separation utilizing a UHPLC Dionex UltiMate 3000 (Thermo Fisher Scientific, Madison, WI, USA) instrument [43]. In the UHPLC, the sample was first trapped with an Acclaim PepMap 100 trap column (100 μm × 2 cm, nanoViper C18, 5 μm, 100 Å) and then washed with 98% solvent A for 6 min at a flow rate of 6 μL/min prior to being separated in an Acclaim PepMap 100 capillary column (75 μm × 15 cm, nanoViper C18, 3 μm, 100 Å) at a flow rate of 400 nL/min. As the UHPLC was running, the LC analytical gradient was increased gradually from 2% to 35% solvent B during the first 90 min and then from 35% to 95% in the next 10 min; finally, 90% and 5% solvent B were run for 5 min and 15 min, respectively. The integration of liquid chromatography–tandem mass spectrometry (LC-MS/MS) with an electrospray ionization source to the quadrupole-based mass spectrometer Q Exactive Orbitrap High-Resolution Mass Spectrometer (Thermo Fisher Scientific, Madison, WI, USA) allowed the resulting peptides to be electro-sprayed through a coated silica emitted tip (PicoTip emitter, New Objective, Massachusetts, USA) at an ion spray voltage of 2000 eV, generating the MS spectra with a resolution of 70,000 (200 *m*/*z*) in a mass range of 350–1800 *m*/*z*. For ion accumulation, 100 ms was set as the maximum injection time. The eluted samples, measured in a data-dependent mode for the 10 most abundant peaks (Top15 method) with the high mass accuracy Orbitrap after ion activation/dissociation with Higher Energy C-trap Dissociation (HCD) at 27 collision energy in a 100–1650 *m*/*z* mass range, were used for MS/MS events (resolution of 17,500) [43]. The obtained proteomics data have been deposited to the ProteomeXchange Consortium via the PRIDE [44] partner repository with the dataset identifier PXD022914.

### 3.4. Data Processing Using MaxQuant Software

The MS spectra of ginseng fruit, leaf, root, and shoot samples were cross-referenced against the genome sequencing database (http://ginsengdb.snu.ac.kr/ (accessed on 1 April 2021)) maintained by Seoul National University [4]. Label-free quantification (LFQ) was performed using default precursor mass tolerances set by Andromeda, with 20 ppm for the first search and 4.5 ppm for the following ones. The search of the LFQ data was based on 0.5 Da of a product mass tolerance with a maximum of two missed tryptic digestions. Carbamidomethylation of cysteine residues was chosen for the fixed modifications, while acetylation of lysine residues and oxidation of methionine residues were selected for additional modifications. The false discovery rate (FDR), which was set at 1% for peptide identifications, was determined based on a reverse nonsense version of the original database.

The data processing for LFQ was performed using MaxLFQ, available as a part of the MaxQuant suite [45]. Subsequently, Perseus software (v. 1.6.14.0) [46] was employed for further statistical and graph analyses. The Perseus software enables performing missing value imputation of protein intensities from a normal distribution (width: 0.3, downshift: 1.8); HCA; and multiple-sample test (one-way ANOVA), controlled by Benjamini–Hochberg method based on an FDR threshold of 0.05, for identifying the significant differences in the protein abundance among the ginseng fruit, leaf, root, and shoot samples. Functional annotation of the identified proteins was undertaken, employing MapMan and AgriGO (v. 2.0) [47,48]. The interaction networks of differentially regulated proteins were predicted by STRING analysis (v. 11.0), coupled with Cytoscape (v. 3.7.2.0) [49]. Subcellular localization analysis was performed using CELLO2GO web-based software [50].

## 4. Conclusions

*P. ginseng* is a precious plant with immense medical and economic value; however, our knowledge about ginseng proteomics is still scanty. Here, a label-free quantitative proteomic analysis was employed to generate a comprehensive proteome map of the ginseng fruit, leaf, root, and shoot. To optimize the extraction of ginseng proteins, we first compared four different protein extraction methods, and we finally adopted the TCA/acetone–MeOH/chloroform method for further analysis. The increased abundance of most of the proteins related to the ginsenoside biosynthesis illustrated that the biosynthesis of ginsenosides in the leaves is probably higher than in the fruit, root, and shoot, while the tissue-specific CYP450s and UGTs might elucidate the existence of proteins specific to each tissue. In addition, the increased abundance of CAS in the shoot and *β*-AS in the leaf leads to speculation that the biosynthesis of sterols might be more active in the shoot samples, whereas the production of oleanane-type ginsenosides might be more active in the leaf tissues. Taken together, the results of the current study show that this efficient and reproducible method for the ginseng protein isolation, which plays a vital role in facilitating the development of ginseng proteomics, provides a relatively comprehensive picture of the ginsenoside biosynthesis and new insights into the protein complement of different ginseng tissues.

## Data Availability

The data presented in this study are available in the article and Supplementary Materials.

## Supplementary Materials

The following are available online at https://www.mdpi.com/article/10.3390/plants10071409/s1, Figure S1: Diagram showing the protein extraction procedures using different protein extraction methods. Figure S2: In-depth proteome analysis of proteins isolated using four different protein extraction methods, namely, TCA/acetone, TCA/acetone–MeOH/chlo, Phenol–TCA/acetone, Phenol–MeOH/chlo. Table S1: Label-free proteomic analysis of proteins isolated from ginseng leaves using TCA/acetone, TCA/acetone–MeOH/chlo, Phenol–TCA/acetone, and Phenol–MeOH/chlo methods, Table S2: Label-free proteomic analysis of proteins isolated from ginseng fruit, leaf, root, and shoot tissues using TCA/acetone–MeOH/chlo, Table S3: Gene ontology (GO) enrichment analysis of proteins isolated from ginseng fruit, leaf, root, and shoot tissues, Table S4: Cell function and metabolism overview of proteins specific to fruit, leaf, root, and shoot using MapMan software, Table S5: List of proteins involved in ginsenoside biosynthesis.
