# Supplementary material for: Optimization of Protein Isolation and Label-Free Quantitative Proteomic Analysis in Four Different Tissues of Korean Ginseng
Source: Plants (Basel). 2021 Jul 9;10(7):1409. doi: 10.3390/plants10071409 (PMC8309323; doi:10.3390/plants10071409)
Supplement: Supplementary file 1 [file plants-10-01409-s001.zip › Figure S1 and S2.pdf]

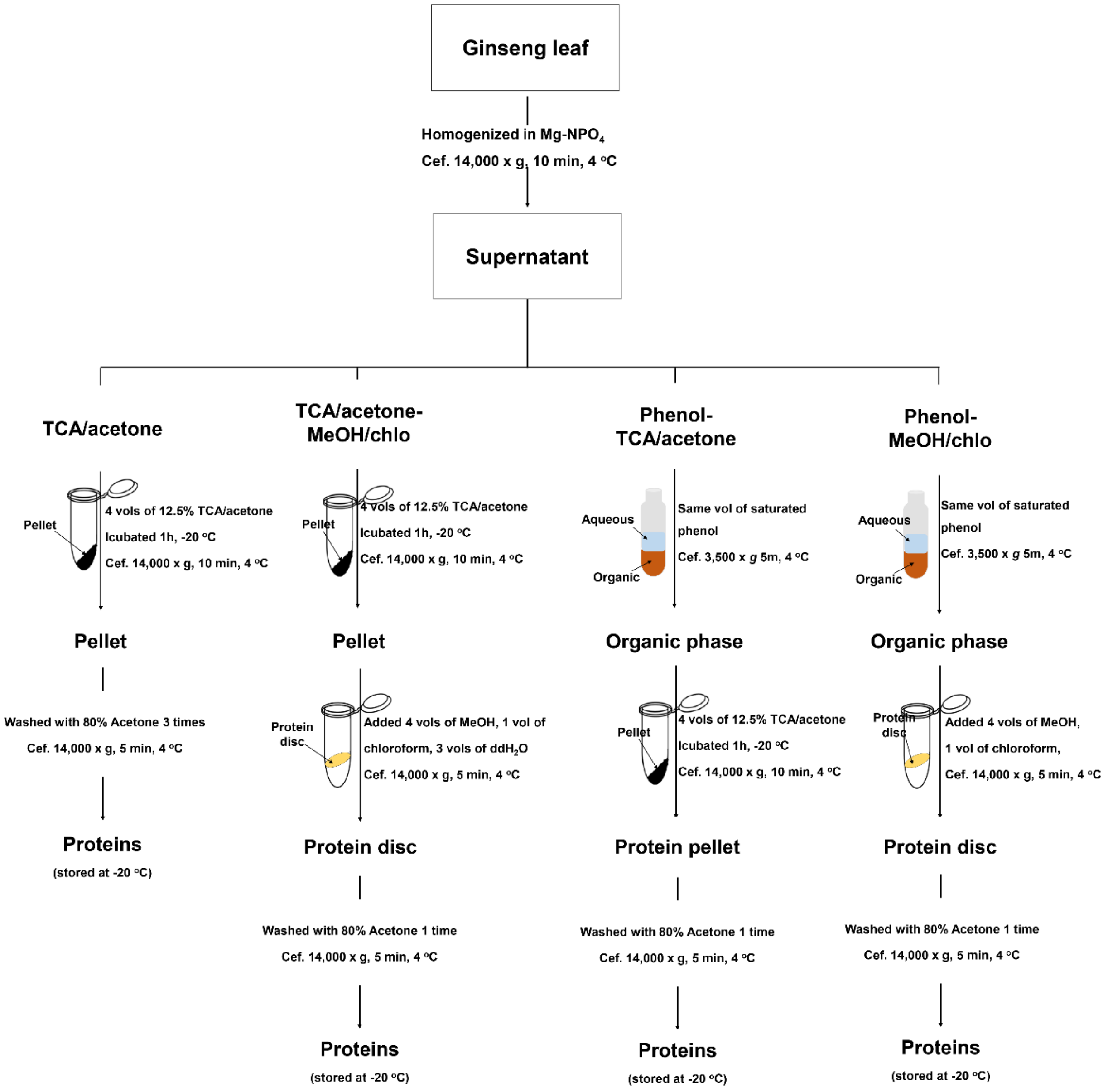

Figure S1. Diagram showing the protein extraction procedures using different protein extraction methods.

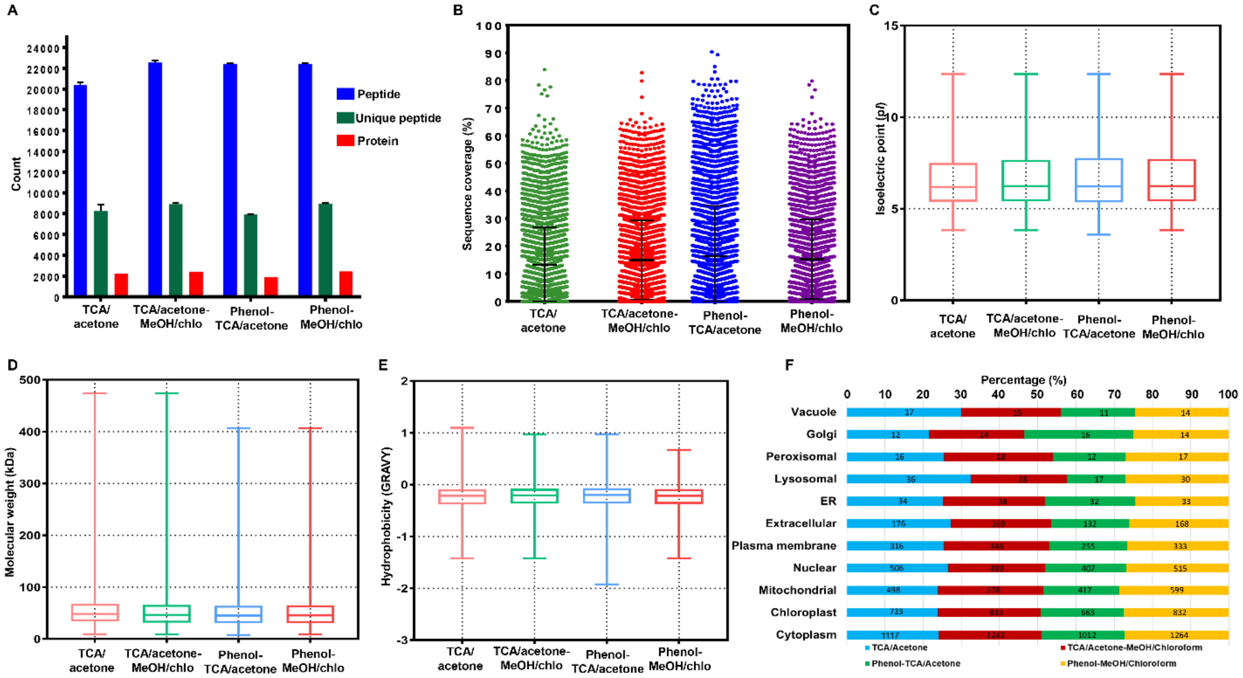

Figure S2. In-depth proteome analysis of proteins isolated using four different protein extraction methods, namely, TCA/acetone, TCA/acetone-MeOH/chlo, Phenol-TCA/acetone, Phenol-MeOH/chlo. The number of identified peptides, unique peptides, and proteins (A). Sequence coverage (B), Isoelectric point (C), Molecular weight (D), Hydrophobicity (E) of identified proteins. Subcellular localization prediction of identified proteins using CELLO2GO web-based software (F).
